# Supplementary material for: Matrix-associated extracellular vesicles modulate smooth muscle cell adhesion and directionality by presenting collagen VI
Source: bioRxiv. 2025 Jul 10:2023.08.17.551257. Preprint. [Version 3] doi: 10.1101/2023.08.17.551257 (PMC10462164; doi:10.1101/2023.08.17.551257)
Supplement: Supplement 13 [file NIHPP2023.08.17.551257v3-supplement-13.pdf]

bioRxiv preprint doi: https://doi.org/10.1101/2023.08.17.551257; this version posted July 10, 2025. The copyright holder for this preprint (which was not certified by peer review) is the author/funder, who has granted bioRxiv a license to display the preprint in perpetuity. It is made available under aCC-BY 4.0 International license.

Supplementary Figures

bioRxiv preprint doi: https://doi.org/10.1101/2023.08.17.551257; this version posted July 10, 2025. The copyright holder for this preprint (which was not certified by peer review) is the author/funder, who has granted bioRxiv a license to display the preprint in perpetuity. It is made available under aCC-BY 4.0 International license.

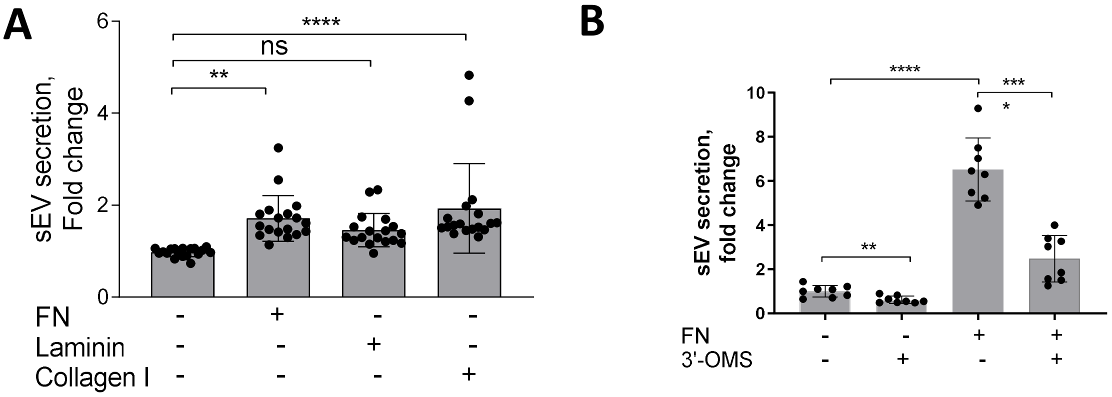

Figure S1. FN matrix stimulates sEV secretion by VSMCs. **A,** FN and collagen I but not laminin stimulate secretion of CD63-enriched sEVs. VSMCs were plated on the various matrices for 24h and sEV secretion was measured by CD63-beads assay. N=3, biological replicates, n=6 technical replicates. ANOVA **p<0.01 **B,** Inhibition of SMPD3 blocks sEV secretion by VSMC plated onto FN matrix. VSMCs were plated on non-coated or FN-coated plates for 24h and conditioned media was analysed by CD63-bead assay. N=2 biological replicates, n=4 technical replicates, ANOVA, ***, p<0.001, ****p<0.0001

bioRxiv preprint doi: https://doi.org/10.1101/2023.08.17.551257; this version posted July 10, 2025. The copyright holder for this preprint (which was not certified by peer review) is the author/funder, who has granted bioRxiv a license to display the preprint in perpetuity. It is made available under aCC-BY 4.0 International license.

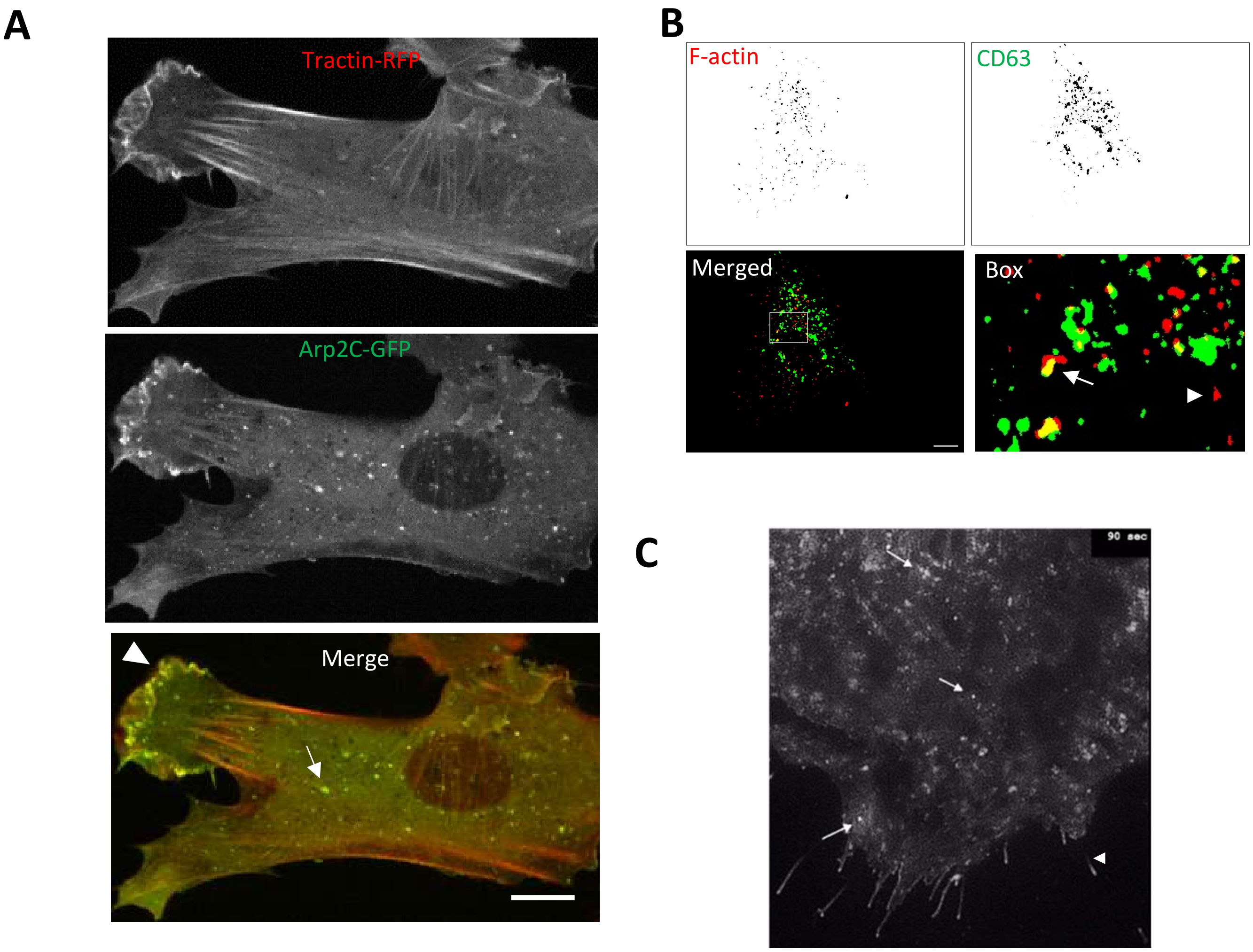

Figure S2. A, Still images of a time-lapse showing that Arp2/3 and F-actin form tails in VSMC cytosol. VSMCs were co-transfected by ARPC2–GFP and F-tractin-RFP and cultured for 24h. Time-lapse video was captured using confocal spinning disk microscopy. Note, that Arp2/3 and F-actin are observed in lamellipodia but also detected in the cytosol with the unknown activity (*arrow*). Size bar, 10µm **B**, Analysis of F-actin/CD63 overlap using ImageJ in a fixed VSMCs. F-actin and CD63 images (Fig 2D) were converted to 8 bits and thresholded. Actin stress fiber structures were extracted from the image by using ParticleAnalysis plug-in (Size (pixel^2) 10-infinity; Circularity 0.5-1) and overlap image created (merged). CD63 endosomes (pseudo coloured in green) were overlapped with F-actin spot-like structures (pseudo coloured in red). Note that endosomes partially overlapped with F-actin tail-like structures (arrows) though some F-actin spots and endosomes were not colocalised (arrowheads).

C, sEV secretion detected with CD63-pHluorin. VSMCs were co-transfected with CD63-pHluorin and incubated for 24h. Time-lapse was captured using confocal spinning-disk microscopy. Arrows, typical "burst"-like appearance of sEV secretion at the cell-ECM interface. Arrows, an intense CD63-pHluorin staining along filopodia-like structures indicating that sEV release can occur in filopodia. Size bar, 10µm

bioRxiv preprint doi: https://doi.org/10.1101/2023.08.17.551257; this version posted July 10, 2025. The copyright holder for this preprint (which was not certified by peer review) is the author/funder, who has granted bioRxiv a license to display the preprint in perpetuity. It is made available under aCC-BY 4.0 International license.

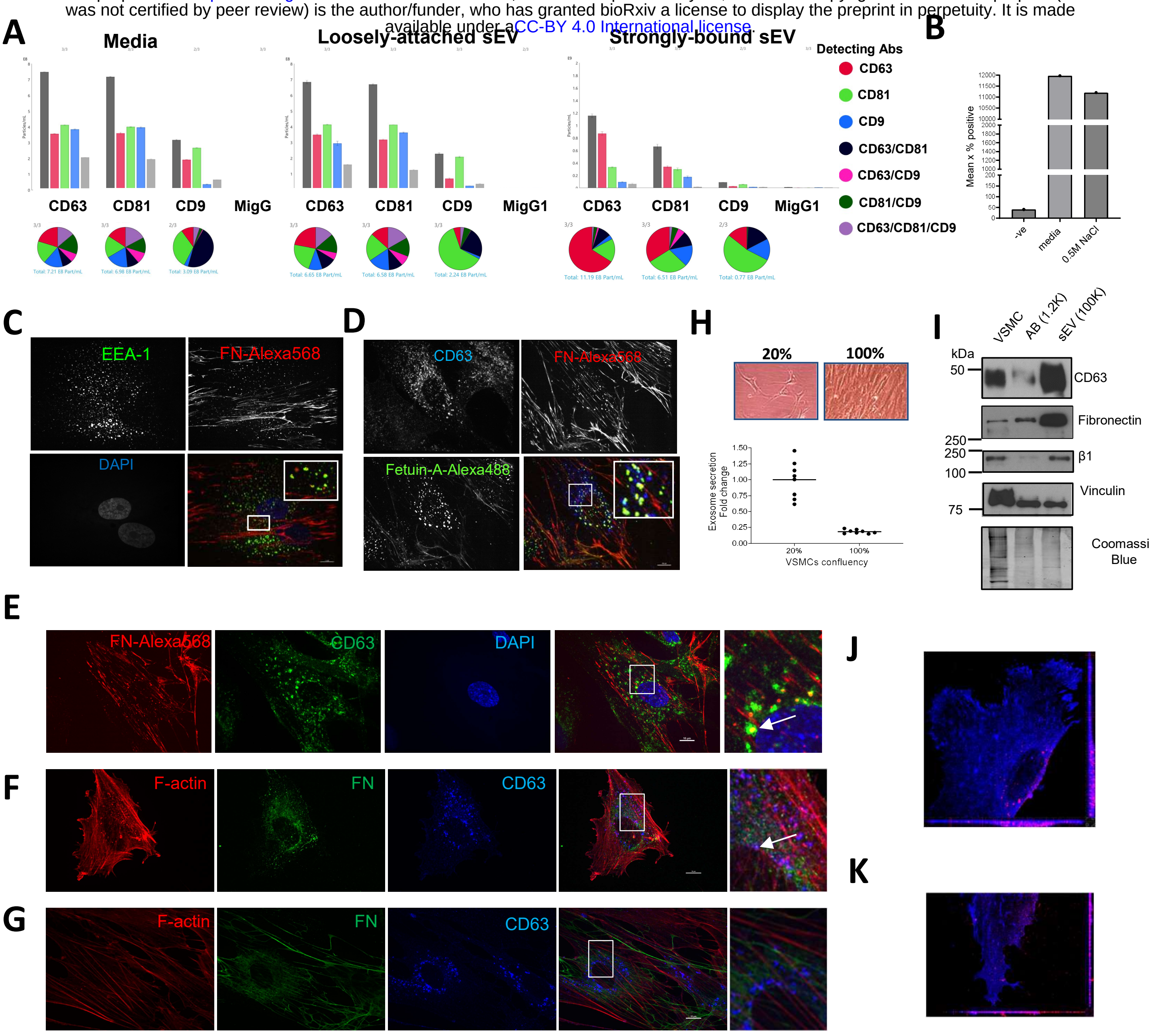

Figure S3 SMPD3-dependent sEVs are trapped in ECM. A, Characterisation of sEVs extracted from VSMC-derived media and ECMs fractions. sEVs were sequentially extracted and fractions were collected and analysed by using ExoView. X-axis – capturing spot antibodies (CD63, CD81, CD9 and control MigG1). **B,** CD63-bead capturing assay shows similar enrichment in CD63+/CD81+ sEVs in the conditioned media and ECM-associated sEVs extracted using 0.5M NaCl. VSMCs were plated onto gelatin-covered plates for 10 days and media was collected every 3d. sEVs were extracted in a mild (0.5M NaCl) conditions and media and 0.5M NaCl fractions were analysed by using CD63-beads capturing assay. **C, D,** Exogenously added FN-Alexa568 can be detected in the early endosomes and MVBs. VSMC were serum-deprived for 24h and then incubated with FN-Alexa568 for 30min (C) or 3h (D) in RPMI-0.5% BSA, thoroughly washed and fixed and stained for EEA-1 (C, green) or CD63 (D, blue). Size bar, 10um, **E,** Exogenously added FN-Alexa568 can be detected in MVBs. VSMC were incubated with FN-Alexa568 for 3h and stained for CD63 which was visualised with anti-mouse-Alexa488 antibody. Size bar, 10um, Note the partial colocalization of FN and CD63. **F, G,** FN can be detected in the CD63+ MVBs in a sparsely growing VSMCs. VSMC were plated at low (5,000 cells per well, **F**) or high density (20,000 cells per well, **G**) and were cultured for 24h in RMPI supplemented with 2.5% EV-free FBS, fixed and stained for F-actin, FN and CD63. **H,** VSMC plating density influences secretion of CD63+/CD81+ sEVs. Cells were plated at the different density and cultured for 24h and sEVs in conditioned media were measured by CD63-beads assay. N=1 biological replicates, n=8 technical replicates. **I**, FN is presented in sEV along with β1 integrin. **J,** VSMC were plated on the FN-coated dishes and Alexa568-labelled sEV were added to the cell media for 3h. Cells were fixed and stained for filopodia marker Myo10 (green) and vinculin (blue). Note perinuclear localisation of internalised sEVs. Size bar, 10µm. 3D projection. Myo10 staining channel is removed. **K**, VSMC were plated on the FN-coated dishes pre-coated with Alexa568-labelled sEV and incubated for 24h. Cell staining as in Fig S3J. Note even distribution of sEVs across the extracellular matrix and cell area.

bioRxiv preprint doi: https://doi.org/10.1101/2023.08.17.551257; this version posted July 10, 2025. The copyright holder for this preprint (which was not certified by peer review) is the author/funder, who has granted bioRxiv a license to display the preprint in perpetuity. It is made available under aCC-BY 4.0 International license.

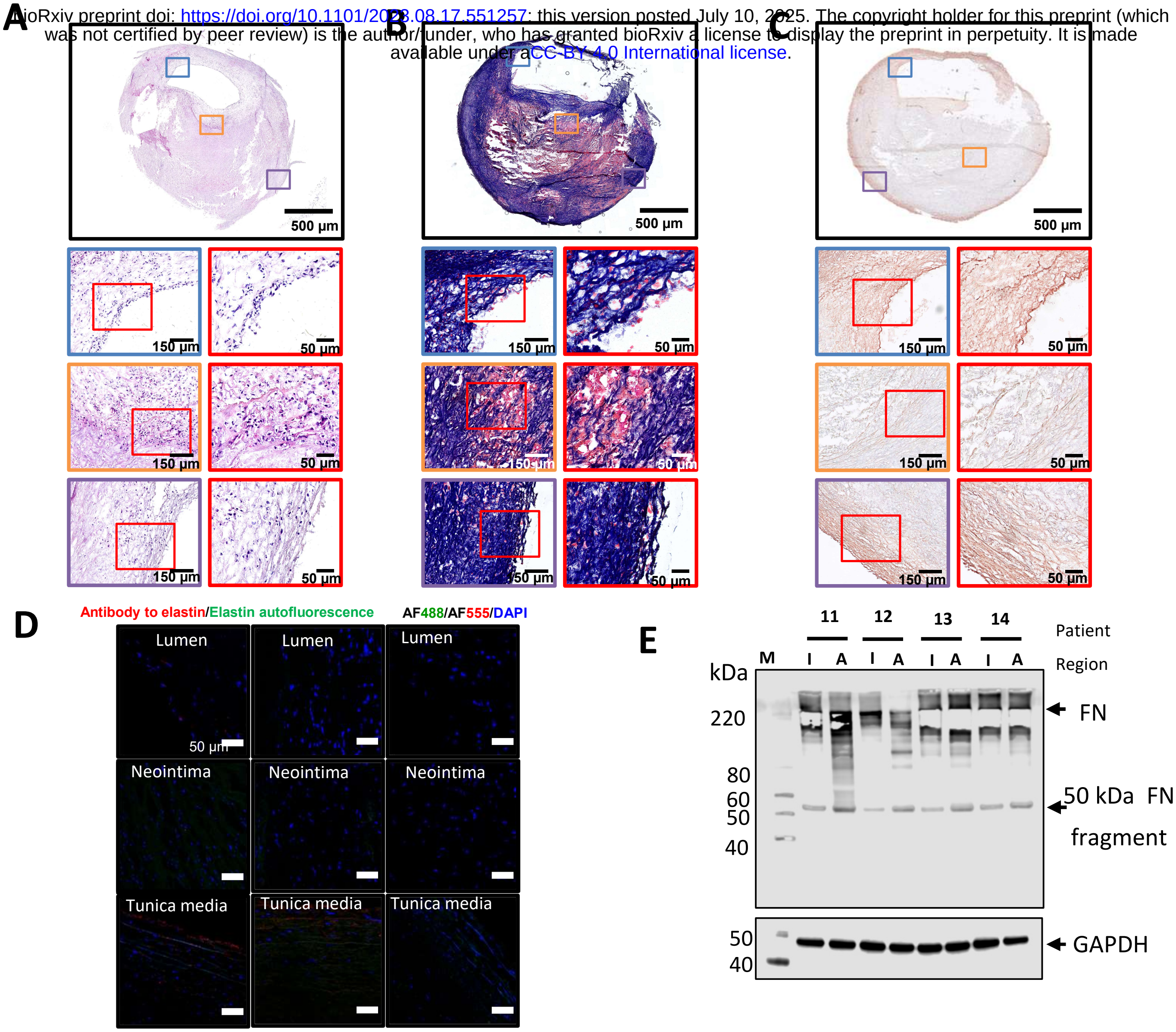

Figure S4. Fibronectin deposition in the atherosclerotic plaque is spatially associated with sEV marker CD81.
A. Staining of atherosclerotic plaques with haematoxylin and eosin. Unstable atheroma with an extracellular lipid or necrotic core and calcification (type IV according to the American Heart Association). Below are representative fields of view focusing on the lumen (blue contour), neointima (green contour), and tunica media (yellow contour). Red contours demarcate representative fields of view within these tissue compartments. Note multiple cells at the luminal side, numerous leukocytes in the neointima, and abundant cells in the tunica media. Overview images: magnification: ×1.8, scale bar: 500 μm; close-ups demarcated by blue, green, and gold contour: magnification: ×200, scale bar, 150 μm; close-ups demarcated by red contour: magnification: ×400, scale bar: 50 μm. **B.** Staining of atherosclerotic plaques with Masson's trichrome. Note the superficial erosions at the luminal side, abundant haemorrhages in the neointima, and sporadic haemorrhages in the tunica media. Overview images: magnification: ×1.8, scale bar: 500 μm; close-ups demarcated by blue, green, and gold contour: magnification: ×200, scale bar: 150 μm; close-ups demarcated by red contour: magnification: ×400, scale bar, 50 μm. **C.** Staining of atherosclerotic plaques with orcein. Note the moderate connective tissue staining at the luminal side, weak connective tissue staining in the atheroma with a confluent extracellular lipid core, and strong specific staining at the tunica media containing elastic fibres. Overview images: magnification: ×1.8, scale bar: 500 μm; close-ups demarcated by blue, green, and gold contour: magnification: ×200, scale bar: 150 μm; close-ups demarcated by red contour: magnification: ×400, scale bar, 50 μm. **D.** Demarcation of tunica media from the neointima using specific antibodies to elastin (red colour) and non-specific autofluorescence of elastic fibers (green colour). Nuclei are counterstained with 4′,6-diamidino-2-phenylindole (DAPI, blue colour). Two representative images per each vascular compartment (luminal side, neointima, and tunica media) are provided. Negative controls (i.e., sections stained with species-specific pre-adsorbed fluorescent-labeled secondary antibodies and DAPI but without the respective antigen-specific primary antibody) are provided to the right from representative images. Magnification: ×200, scale bar, 50 μm. Note that elastin is expressed exclusively in the tunica media and is associated with elastic fibers. **E.** Expression of FN in atherosclerotic plaques. Atherosclerotic plaques (A) and adjacent intact arterial segments (I) were homogenised and analysed by Western blotting. FN is abundantly presented in both regions and 50 kDa FN fragment (Homandberg et al., 1992) intensity was used to quantify FN amount.

bioRxiv preprint doi: https://doi.org/10.1101/2023.08.17.551257; this version posted July 10, 2025. The copyright holder for this preprint (which was not certified by peer review) is the author/funder, who has granted bioRxiv a license to display the preprint in perpetuity. It is made available under aCC-BY 4.0 International license.

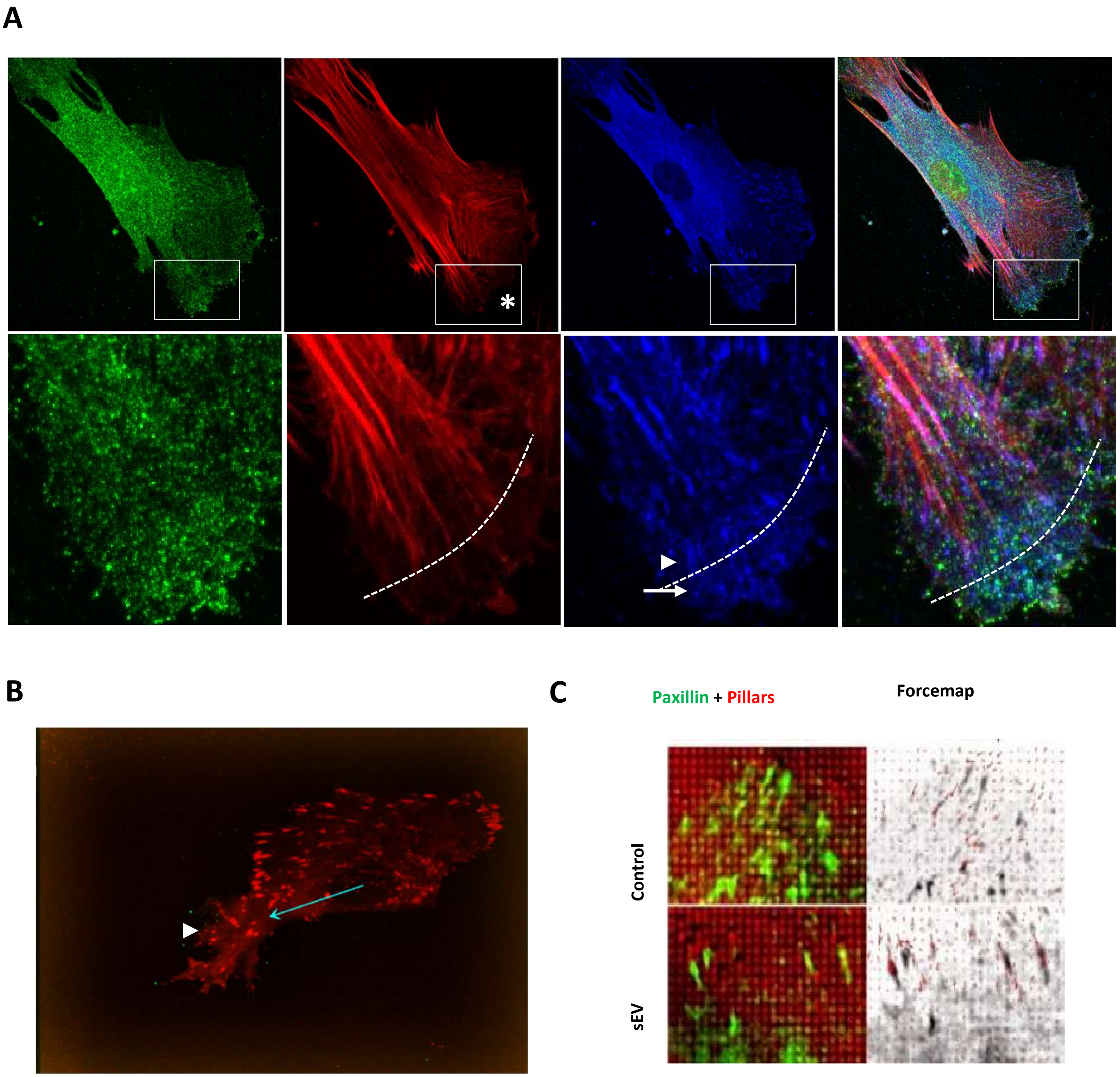

Supplementary Figure S5 sEVs regulate VSMC motility and invasion. A, Centripetal FAs are linked to actin stress fibers. VSMCs were plated on FN-coated plate for 24h and cells were stained for Myo10 (green), CD63 (blue) and F-actin (phalloidin, red). Note the dot-like focal complexes in lamellipodium which are not associated with the contractile actin bundles (arrow) and an appearance of elongated FAs associated with the mature actin bundles (arrowhead). Dotted line, approximate position of the lamellipodium boundaries. Size bar, 10µm. **B,** Mature focal adhesion turnover is not affected by sEVs. VSMC were transfected with Paxillin-RFP and plated on the FN in the absence or presence of immobilised sEVs. Images were captured for 30min using confocal spinning disk microscopy. Note the appearance of the mature FAs in the lamellipodium (Arrowhead). Arrow, direction of the VSMC movement. Representative from N=4 biological replicates. **C,** sEV induces formation of FAs with the enhanced pulling force. VSMC transfected with Paxillin-RFP were plated on the PDMS pillars which were covered with FN and sEVs and pillar displacements were quantified**.** Representative image from N=2 biological replicates.

bioRxiv preprint doi: https://doi.org/10.1101/2023.08.17.551257; this version posted July 10, 2025. The copyright holder for this preprint (which was not certified by peer review) is the author/funder, who has granted bioRxiv a license to display the preprint in perpetuity. It is made available under aCC-BY 4.0 International license.

A

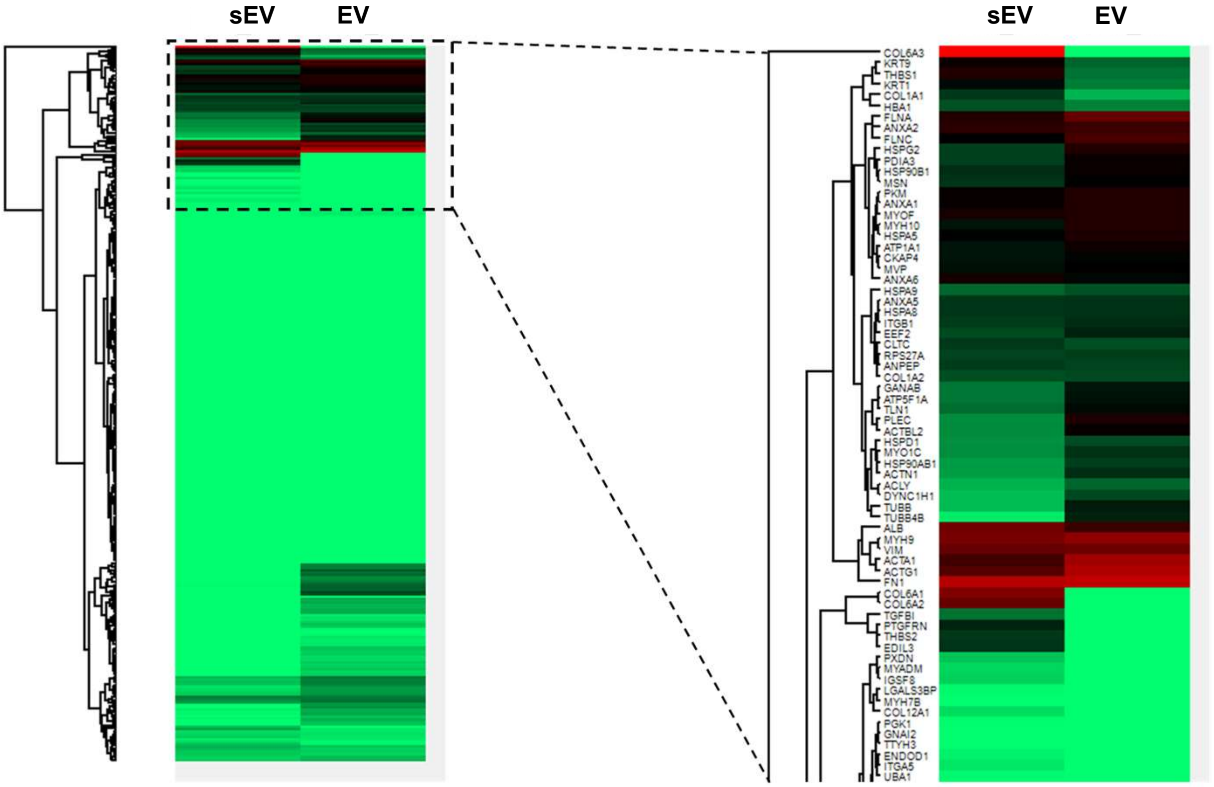

B

Differential Genes	Intensity sEV	Intensity EV	Log2FC (sEV/EV)
COL6A3	23.5557	16.3415	7.2141
KRT9	20.1342	18.8244	1.3098
THBS1	20.3928	18.6476	1.7451
KRT1	19.8617	18.5279	1.3339
COL1A1	19.3296	17.8776	1.4520
HBA1	19.0504	18.4673	0.5831
FLNA	20.3898	21.1759	-0.7861
ANXA2	20.4767	20.6856	-0.2089
FLNC	20.0150	20.8167	-0.8018
HSPG2	19.2403	20.3940	-1.1537
PDIA3	19.2507	20.1260	-0.8754
HSP90B1	19.4555	20.0967	-0.6412
MSN	19.3903	19.9811	-0.5908
PKM	20.0832	20.3937	-0.3105
ANXA1	20.0844	20.3684	-0.2839
MYOF	20.2715	20.3869	-0.1154
MYH10	19.7632	20.3833	-0.6201
HSPA5	19.9596	20.3367	-0.3771
ATP1A1	19.7624	20.1765	-0.4141
CKAP4	19.7636	19.9494	-0.1858
MVP	19.8283	20.0128	-0.1845
ANXA6	20.2369	19.9185	0.3185
HSPA9	18.7952	19.0373	-0.2421
ANXA5	19.3647	19.4175	-0.0528
HSPA8	19.3616	19.4092	-0.0476
ITGB1	19.2970	19.4810	-0.1840
EEF2	19.1407	19.6215	-0.4808
CLTC	19.3425	19.0681	0.2745
RPS27A	19.2068	19.2699	-0.0631
ANPEP	19.2804	19.2108	0.0696
COL1A2	19.0642	19.1503	-0.0861
GANAB	18.6016	19.7384	-1.1368
ATP5F1A	18.6089	19.7824	-1.1735
TLN1	18.7263	19.8785	-1.1522
PLEC	18.3459	20.3391	-1.9932
ACTBL2	18.3658	20.0872	-1.7214
HSPD1	18.3079	19.1233	-0.8154
MYO1C	18.3590	19.4099	-1.0508
HSP90AB1	18.1685	19.2795	-1.1110
ACTN1	18.1416	19.4682	-1.3266
ACLY	17.9105	18.8048	-0.8943
DYNC1H1	17.7699	19.1439	-1.3741
TUBB	17.7978	19.6994	-1.9017
TUBB4B	17.2412	19.6159	-2.3747
ALB	21.3341	20.6810	0.6531
MYH9	21.4046	21.6826	-0.2780
VIM	21.1810	21.2746	-0.0937
ACTA1	20.7441	21.8970	-1.1529
ACTG1	20.9511	22.0509	-1.0998
FN1	22.1092	22.2403	-0.1311
COL6A1	21.4999	16.5122	4.9877
COL6A2	21.2127	15.6383	5.5744
TGFBI	18.6599	15.4468	3.2131
PTGFRN	19.5851	15.6142	3.9709
THBS2	19.3408	15.6576	3.6832
EDIL3	19.3888	15.4599	3.9289

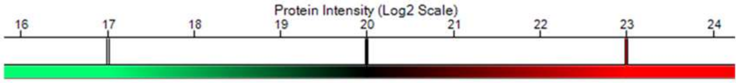

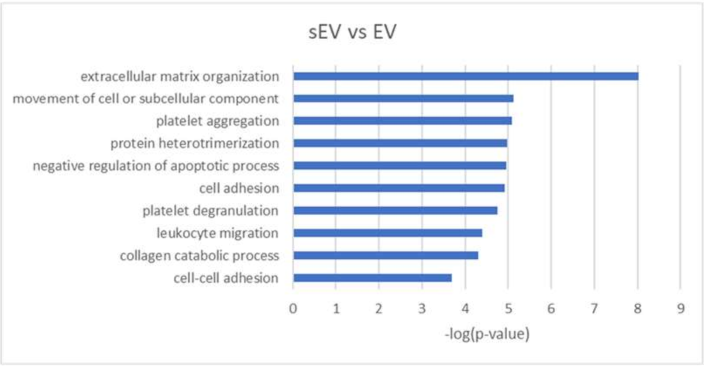

Supplementary Figure S6. Proteomic analysis of VSMC-derived sEVs and EV. VSMC-derived EVs and sEVs were isolated from cells and analyzed by protein mass-spectrometry. N=3 biological replicates. **A.** Clustered proteomic heatmap for EV and sEV. **B,** The GO term analysis was performed using the differentially expressed genes between the sEV and EV groups.
