# Supplementary material for: Extracellular vesicles stimulate smooth muscle cell migration by presenting collagen VI
Source: bioRxiv. 2023 Aug 22:2023.08.17.551257. Preprint. [Version 2] doi: 10.1101/2023.08.17.551257 (PMC10462164; doi:10.1101/2023.08.17.551257)
Supplement: Supplementary file 1 [file NIHPP2023.08.17.551257v2-supplement-13.pdf]

Supplementary Figures

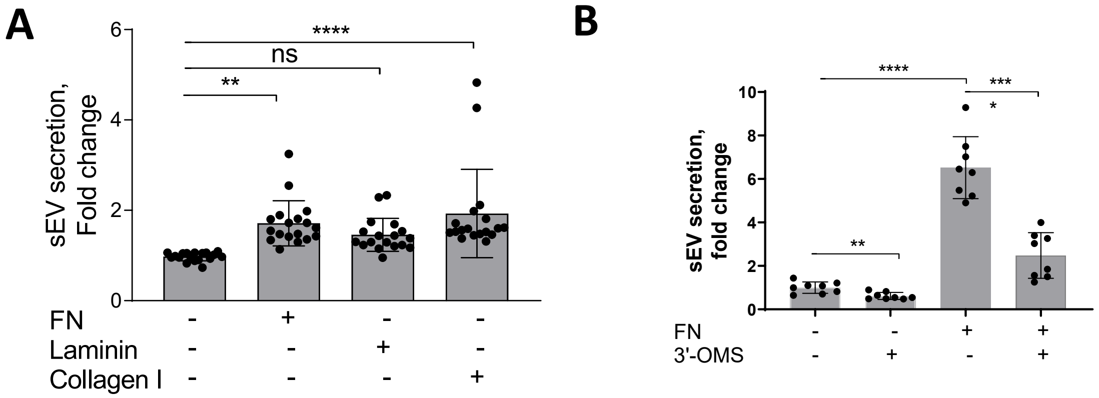

Figure S1. FN matrix stimulates sEV secretion by VSMCs. **A,** FN and collagen I but not laminin stimulate secretion of CD63-enriched exosomes. VSMCs were plated on the various matrices for 24h and sEV secretion was measured by CD63-beads assay. N=3, ANOVA **p<0.01 **B,** Inhibition of SMPD3 blocks sEV secretion by VSMC plated onto FN matrix. VSMCs were plated on non-coated or FN-coated plates for 24h and conditioned media was analysed by CD63-bead assay. N=2 with n=4 for each, ANOVA, ***, p<0.001, ****p<0.0001

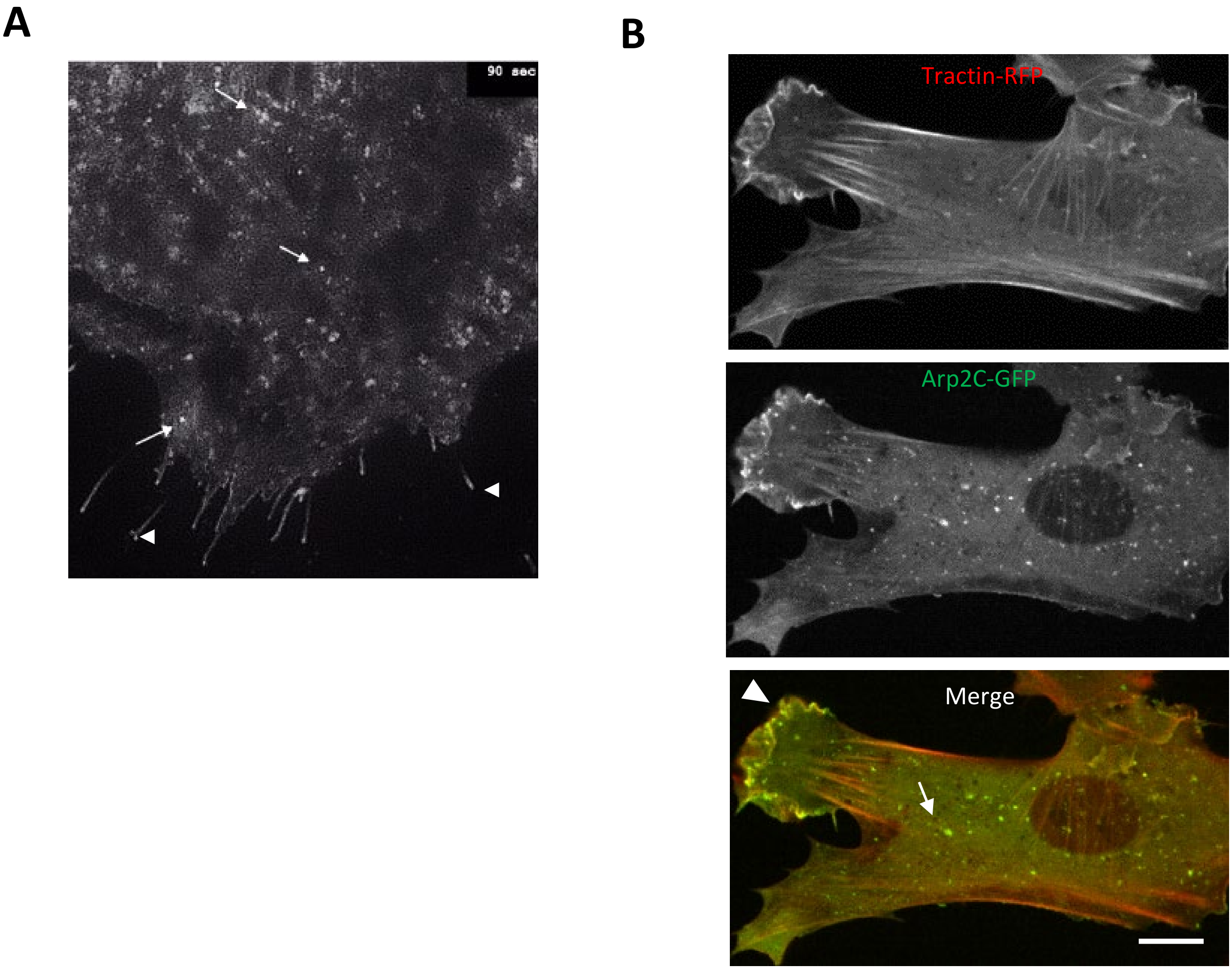

Supplementary Figure S2 A, sEV secretion detected with CD63-pHluorin. VSMCs were co-transfected with CD63-pHluorin and incubated for 24h. Time-lapse was captured using confocal spinning-disk microscopy. Arrows, typical “burst”-like appearance of sEV secretion at the cell-ECM interface. Arrows, an intense CD63-pHluorin staining along filopodia-like structures indicating that sEV release can occur in filopodia. **B**, Still images of a time-lapse showing that Arp2/3 and F-actin form tails in VSMC cytosol. VSMCs were co-transfected by ARPC2–GFP and F-tractin-RFP and cultured for 24h. Time-lapse video was captured using confocal spinning disk microscopy. Note, that Arp2/3 and F-actin are observed in lamellipodia but also detected in the cytosol with the unknown activity (*arrow*). Size bar, 10µm

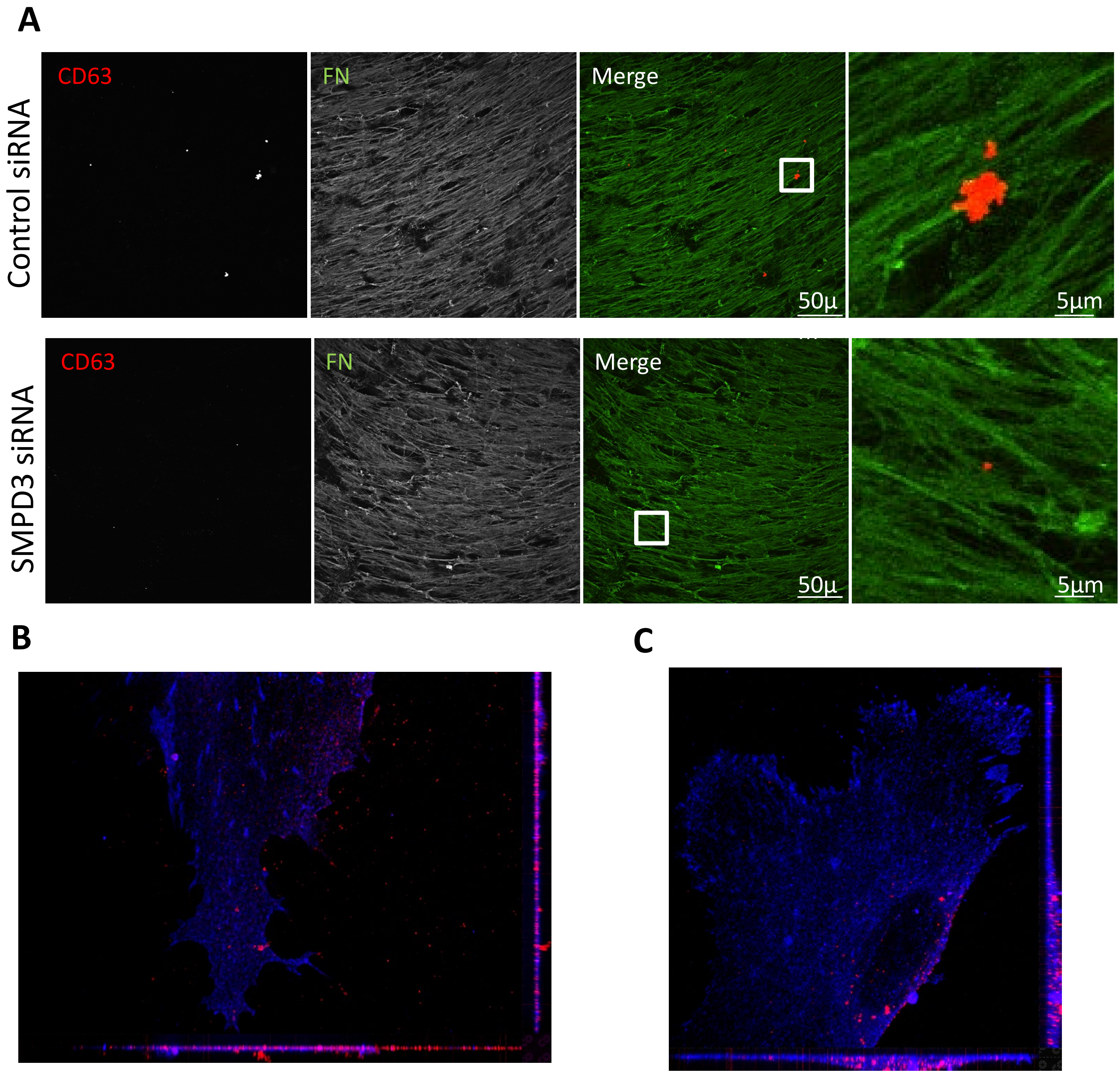

Figure S3 SMPD3-dependent sEVs are trapped in ECM. A. VSMCs were plated onto gelatin-covered plates and treated with control or SMPD3 siRNA for 72 hrs. 3D matrices were generated as described in "Materials and methods" and stained for CD63 and fibronectin. Images were acquired using Nikon AX inverted confocal microscope. Oil 60x Objective. Note the decrease in CD63-positive sEVs associated with the FN fibrils.
B. VSMC were plated on the FN-coated dish and Alexa568-labelled sEV were added to the cell media for 3 h. Cells were fixed and stained for filopodia marker Myo10 (green) and vinculin (blue). Note perinuclear localisation of internalised sEVs. Size bar, 10µm. 3D projection. Myo10 staining channel is removed. Representative image from N=3. **C**, VSMC were plated on the FN-coated dish pre-coated with Alexa568-labelled sEV and incubated for 24h. Cell staining as in Fig 3B. Note even distribution of sEVs across the extracellular matrix and cell area.

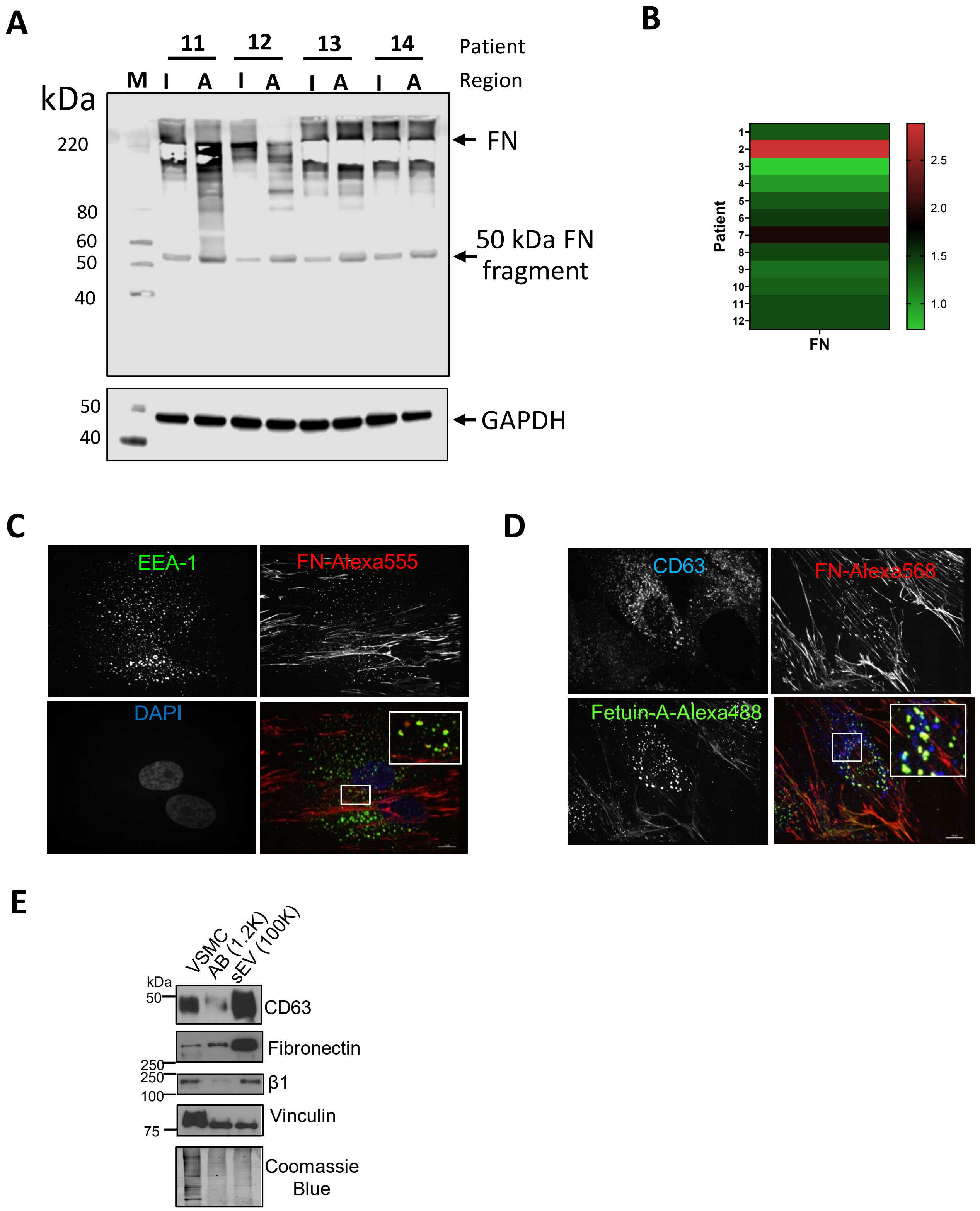

Figure S4. Fibronectin deposition in the atherosclerotic plaque is spatially associated with sEV marker CD81. A. Expression of FN in atherosclerotic plaques. Atherosclerotic plaques (A) and adjacent intact arterial segments (I) were homogenised and analysed by Western blotting. FN is abundantly presented in both regions and 50 kDa FN fragment (Homandberg et al., 1992) intensity was used to quantify FN amount. Representative image for N=4 **B**. Quantification of FN content in atherosclerotic plaques. Heatmap for figure S4A. **D, E,** Exogenously added FN-Alexa555 can be detected in the early endosomes and MVBs. VSMC were incubated with FN-Alexa555 for 30min (D) or 3h (E) and stained for EEA-1 (D, green) or CD63 (E, blue). Size bar, 10um, **F,** FN is presented in sEV along with β1 integrin. Western blot analysis of isolated VSMC-derived sEVs.

A

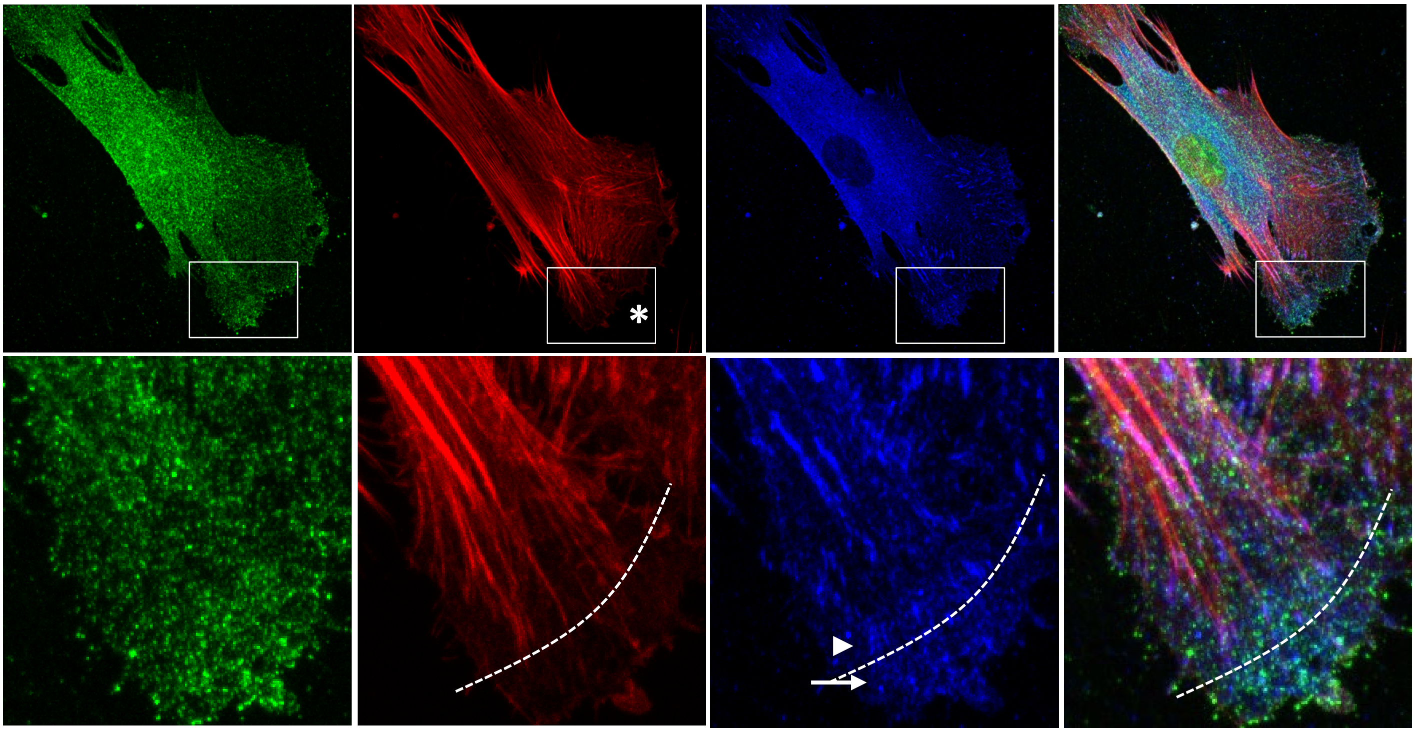

B

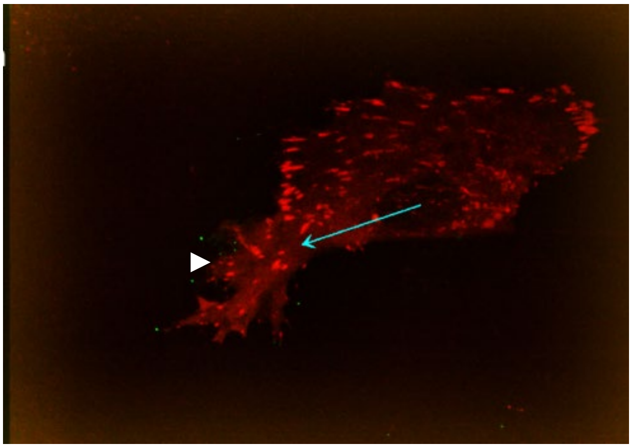

C

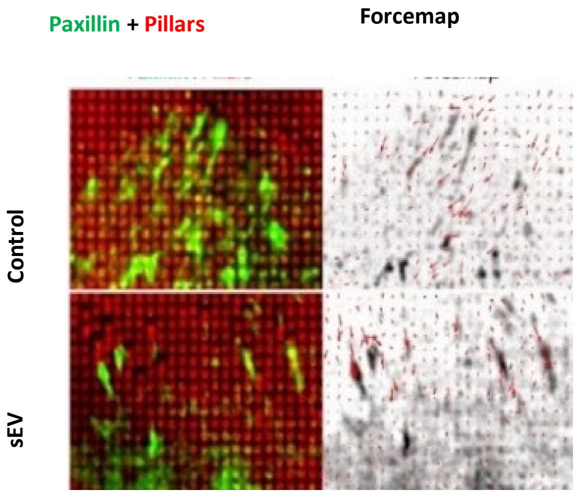

Supplementary Figure S5 sEVs regulate VSMC motility and invasion. A, Centripetal FAs are linked to actin stress fibers. VSMCs were plated on FN-coated plate for 24h and cells were stained for Myo10 (green), CD63 (blue) and F-actin (phalloidin, red). Note the dot-like focal complexes in lamellipodium which are not associated with the contractile actin bundles (arrow) and an appearance of elongated FAs associated with the mature actin bundles (arrowhead). Dotted line, approximate position of the lamellipodium boundaries. Size bar, 10µm. **B,** Mature focal adhesion turnover is not affected by sEVs. VSMC were transfected with Paxillin-RFP and plated on the FN in the absence or presence of immobilised sEVs. Images were captured for 30min using confocal spinning disk microscopy. Note the appearance of the mature FAs in the lamellipodium (Arrowhead). Arrow, direction of the VSMC movement. N=4. **C,** sEV induces formation of FAs with the enhanced pulling force. VSMC transfected with Paxillin-RFP were plated on the PDMS pillars which were covered with FN and sEVs and pillar displacements were quantified**.** Representative image from N=2.

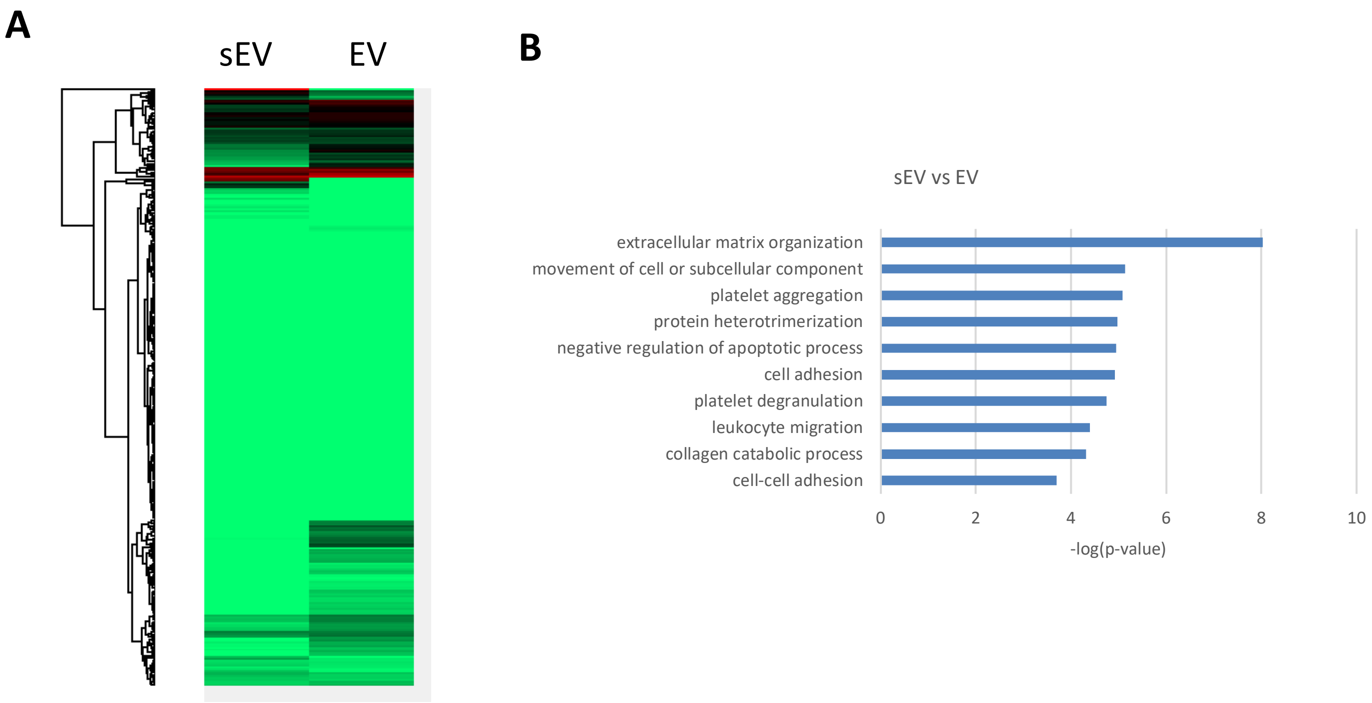

Supplementary Figure S6. Proteomic analysis of VSMC-derived sEVs and EV. VSMC-derived EVs and sEVs were isolated from cells and analyzed by protein mass-spectrometry. N=3. **A.** Clustered proteomic heatmap for EV and sEV. **B,** GO functional enrichment analysis
